# Radioembolization Followed by Transarterial Chemoembolization in Hepatocellular Carcinoma

**DOI:** 10.7759/cureus.23783

**Published:** 2022-04-03

**Authors:** Baran U Vardar, Ece Meram, Kerim Karaoglu, Muxuan Liang, Menggang Yu, Paul Laeseke, Orhan S Ozkan

**Affiliations:** 1 Radiology, University of Wisconsin School of Medicine and Public Health, Madison, USA; 2 Biostatistics and Medical Informatics, University of Wisconsin, Madison, USA

**Keywords:** liver dysfunction, toxicity, chemoembolization, hepatocellular carcinoma, radioembolization

## Abstract

Background and objective: In recent years, combination therapies for hepatocellular carcinoma (HCC) have been increasingly used with superior treatment responses compared to monotherapies. However, the safety and efficacy of the transarterial chemoembolization (TACE) and transarterial radioembolization (TARE) combinations for HCC patients have not been investigated in the literature. In this study, our aim was to evaluate the safety and outcomes of TACE after TARE in HCC patients.

Materials and methods: All TARE procedures performed on HCC patients at a single institution between January 2008 and November 2016 were retrospectively reviewed. Seventy-three patients who did not receive any additional transarterial therapy in the areas targeted by TARE were assigned to the “TARE group,” while 27 patients who received TACE after TARE to the same target area were assigned to the “Combo group.” Post-procedural liver toxicity, tumor response, overall survival (OS), and time to progression (TTP) were evaluated.

Results: Fewer patients in the Combo group had worsening liver function than the TARE group based on the change in bilirubin levels (19% vs. 40%; p=0.029) and Child-Pugh score increase (28% vs. 51%; p=0.056). The median OS time of all patients was 11.04 months. The Combo group had a significantly longer median OS of 36.8 months (vs. 10.6, p=0.003) and median TTP of 14.4 months (vs. 5.5, p=0.018). After accounting for selection bias, OS and TTP were still in favor of the Combo group, with hazard ratios of 0.651 (p<0.05) and 0.63 (p<0.05), respectively.

Conclusion: The addition of TACE to TARE is a safe and effective treatment in unresectable HCC patients and can be considered in select patients with a lack of complete response or disease progression.

## Introduction

Hepatocellular carcinoma (HCC) is the most common primary malignancy of the liver, and its global incidence and mortality are rising, especially in the United States and Europe [1]. Based on the Barcelona Clinic Liver Cancer (BCLC) classification, curative treatments such as liver transplantation, surgical resection, and local ablation are recommended for early-stage HCC patients [2]. However, HCC is often diagnosed at an intermediate or advanced stage when the patients are no longer eligible for curative options [2]. For intermediate stage (BCLC stage B) patients with sufficient liver function, transarterial chemoembolization (TACE) is recommended as a standard of care by the American Association for the Study of Liver Diseases (AASLD) and the European Association for the Study of the Liver (EASL) guidelines [2-4].

Another transarterial treatment method, transarterial radioembolization (TARE) with Y90-coated microspheres, was shown in multiple retrospective studies to produce similar time-to-progression (TTP) and overall survival (OS) periods compared to TACE in patients with unresectable HCC [5,6]. Salem et al. showed that the TTP of patients treated with TARE was significantly longer in a prospective, randomized study comparing radioembolization with conventional TACE [7]. TARE was reported to be used as a first-line transarterial treatment for patients with unresectable HCC and good liver function in certain centers [8].

The combination of TACE and external radiotherapy is suggested to have synergistic effects and may remedy the limitation of each alone in HCC patients [9,10]. However, the addition of TACE to radioembolization as a selective internal radiation treatment has not yet been extensively studied. The purpose of this study was to retrospectively review the treatment outcomes, safety profiles, and failure patterns of TARE followed by TACE in patients with intermediate or advanced stage HCC.

## Materials and methods

Patient selection

This Health Insurance Portability and Accountability Act (HIPAA) compliant study was conducted with the institutional review board's approval. Patients with HCC who underwent TARE at our institution from January 2008 to November 2016 were retrospectively reviewed. The diagnosis of HCC was confirmed either by a biopsy or based on the imaging criteria of the AASLD guidelines [2,3]. The patients provided written informed consent.

In the study, patients with unresectable HCC were included if they had a BCLC disease stage of B or C, a Child-Pugh (CP) Class A or B, an Eastern Cooperative Oncology Group (ECOG) performance status of 0 or 1, and no prior locoregional transarterial treatment to TARE-targeted areas.

After the database review, 100 patients were identified. Seventy-three patients who did not receive any additional transarterial therapy to the areas targeted by TARE were assigned to the “TARE group,” while 27 patients who received TACE after TARE for the treatment of the same target lesion/area were assigned to the “Combo group.” Because TARE does not preclude subsequent intra-arterial therapies owing to its minimal embolic effect, in some patients who had a lack of complete response or a progression in TARE targeted areas, TACE was considered as a combination therapy for TARE-targeted areas. Any TACE procedure performed for an area other than the TARE-targeted area was recorded as an additional therapy but was not included in the Combo group.

Patient demographics and baseline characteristics were recorded. Patients were classified into nominal or ordinal groups based on previously reported cut-off values of serum alpha-fetoprotein (AFP) levels and model for end-Stage Liver Disease (MELD) scores [11,12]. For each patient, the treatment modality, the number of treatment sessions, and the decision of single lobe or whole liver treatment were determined by a multidisciplinary tumor board comprising medical oncologists, surgeons, hepatologists, and interventional radiologists.

Transarterial radioembolization

All patients underwent an arteriogram to delineate the hepatic arterial anatomy, identify tumoral and possible extrahepatic perfusion, and estimate lung shunt fraction based on Tc-99m macroaggregated albumin scintigraphy. The radiation dose to be delivered was calculated based on the target liver volume per the manufacturer's guidelines. On a later day, patients received TARE with Y-90 coated glass microspheres (TheraSphere, BTG International, West Conshohocken, PA). Lobar treatments were used in 72 patients; two segments were targeted in 17 patients, and one segment was targeted in 11 patients. Overall, 134 TARE procedures were performed in 100 patients with a median radiation dose of 120 Gy (range, 75-135 Gy).

Transarterial chemoembolization

Conventional TACE (cTACE) was performed using emulsification of ethiodized oil, water-soluble contrast material, 30 mg of doxorubicin, 10 mg of mitomycin C, and 100 mg of cisplatin. It was followed by an injection of 1-2 ml of 100-300 micron particles depending on the operator preference and the presence of forwarding flow at the completion of the infusion. Drug-eluting bead TACE (DEB-TACE) procedures were performed using LC Bead™ (Biocompatibles UK Ltd, Farnham, UK) mixed with 75-125 mg of doxorubicin.

TACE was performed after TARE for patients with sufficient liver reserve, either early in the follow-up when there was a lack of complete response or later when disease progression was observed. Thirty-five cTACE, eight DEB-TACE, and five bland embolization procedures were performed in the Combo group.

Follow-up imaging and response evaluation

The median follow-up time for all patients calculated with the reverse Kaplan-Meier estimator was 34.9 months (95% CI: 25.1-44.7). Cross-sectional imaging modalities were obtained at a median of 45 days (range between 21 days and 99 days) after the procedure and then once every three months. After the procedure, imaging modality selection (CT or MRI) was decided based on the imaging modality used before the procedure and treatment-specific factors such as the use of Lipiodol. Contrast-enhanced MRI was used as the primary imaging modality in cases that received Lipiodol.

The follow-up imaging studies were retrospectively reviewed by two attending interventional radiologists (20 and five years of experience) in two separate picture archiving and communication system (PACS) stations. Treatment response was evaluated using modified Response Evaluation Criteria in Solid Tumors (mRECIST) [13].

Toxicity

Worsening liver function based on the patients' biochemical and clinical findings was defined as a persistent increase in total serum bilirubin of more than 2 mg/dL or a rise of at least two points in Child-Pugh (CP) score compared to pre-procedure values within 12 months after the last procedure [14,15]. Patients' laboratory results were recorded at three, six, and 12 months after the first TARE procedure in the TARE group and after the first TACE procedure in the Combo group. Of the 100 patients, 12 patients lacked the necessary clinical and laboratory follow-up information for CP calculation, and these patients were excluded from the statistical analysis of toxicity assessment. Major adverse events and complications were noted based on the Society of Interventional Radiology (SIR) grading for procedural complications [16]. Cancer Therapy Evaluation Program's Common Terminology Criteria for Adverse Events (CTCAE) version 4.0 was taken into consideration for toxicity assessment [5,17].

Statistical analysis

For testing the selection bias of baseline features between two groups, Fisher's exact test was used for categorical variables, and a simple t-test was used for continuous variables.

Analyses of OS and TTP were performed using Kaplan-Meier curves and log-rank test underweighted Cox proportional hazards model for which propensity score weighting was applied to avoid selection bias caused by baseline characteristics. In the fitting procedure of the propensity score, all recorded variables in the study were incorporated. Age and tumor distribution were excluded because they did not have a large impact on the cohort balancing after the adjustment. Also, cirrhosis and other variables with low prevalence were excluded to ensure a stable estimation of the propensity score.

Median TTP and OS were calculated from the date of the first TARE treatment. For TTP and OS calculations, we censored patients on the date of death, last follow-up, or liver transplantation. P-values of less than 0.05 were regarded as statistically significant. All analyses were conducted using R software version 4.1.0 (R Core Team, Vienna, Austria).

## Results

Baseline characteristics

The TARE and Combo groups had similar baseline characteristics in age, gender, Child-Pugh score, MELD score, AFP value, etiology of liver disease, tumor distribution, percentage of patients with cirrhosis, portal vein tumor thrombus (PVTT), and extrahepatic metastases (Tables 1, 2). However, the Combo group had a higher percentage of BCLC stage-B (p=0.015), ECOG 0 (p=0.043), and solitary tumors (p=0.005). The longest diameter of the target lesions' enhancing regions was between 1.7 and 15.5 cm with a median value of 5.0 cm (standard deviation of 2.88 cm).

**Table 1:** Baseline patient demographics of the TARE and Combo groups. Note: MELD, Model for End-Stage Liver Disease; BCLC, Barcelona Clinic Liver Cancer classification; ECOG, Eastern Cooperative Oncology Group; AFP, serum alpha-fetoprotein levels; PVTT, portal vein tumor thrombus; TACE, transarterial chemoembolization.

Variable		Overall n=100 (%)	TARE n=73 (%)	Combo n=27 (%)	
					Sex					
	Male	88 (88)	63 (86)	25 (93)	
	Female	12 (12)	10 (14)	2 (7)	
Age		64.5 (38-90)	64.3 (45-90)	65.1 (38-83)	
Etiology					
	HCV	39 (39)	31 (42)	8 (30)	
	Alcohol	22 (22)	17 (23)	5 (19)	
	Cryptogenic	10 (10)	8 (11)	2 (7)	
	NASH	7 (7)	5 (7)	2 (7)	
	HBV	6 (6)	2 (3)	4 (15)	
	Other	16 (16)	10 (14)	6 (22)	
Cirrhosis		87 (87)	66 (90)	21 (78)	
Child-Pugh					
	A	75 (75)	53 (73)	22 (81)	
	B	25 (25)	20 (27)	5 (19)	
MELD					
	≤9	65 (65)	45 (62)	20 (74)	
	>9	35 (35)	28 (38)	7 (26)	
BCLC		0			
	B	32 (32)	18 (25)	14 (52)	
	C	68 (68)	55 (75)	13 (48)	
ECOG					
	0	42 (42)	26 (36)	16 (59)	
	1	56 (56)	46 (63)	10 (37)	
	2	2 (2)	1 (1)	1 (4)	
Tumor Type					
	Solitary	14 (14)	5 (7)	9 (33)	
	Multifocal	58 (58)	46 (63)	12 (44)	
	Infiltrative	28 (28)	22 (30)	6 (22)	
Tumor Distribution					
	Unilobar	36 (36)	24 (33)	12 (45)	
	Bilobar	64 (64)	49 (67)	15 (55)	
AFP					
	>400	32 (32)	26 (36)	6 (22)	
PVT					
	No	68 (68)	48 (66)	20 (74)	
	Yes	32 (32)	25 (34)	7 (26)	
Extrahepatic Metastasis					
	No	96 (96)	70 (96)	26 (96)	
	Yes	4 (4)	3 (4)	1 (4)	
Prior Treatment					
	Prior Surgery	14 (14)	11 (15)	3 (11)	
	Prior Ablation	17 (17)	16 (22)	1 (4)	
	Prior Chemotherapy	9 (9)	7 (10)	2 (7)	
Additional Treatment					
	Additional Ablation	5 (5)	4 (5)	1 (4)	
	Additional Surgery	2 (2)	2 (2)	0 (0)	
	Additional Chemotherapy	12 (12)	6 (8)	6 (22)	
	Additional TACE	18 (18)	7 (10)	11 (41)	
Sorafenib Use		16 (16)	10 (14)	6 (22)	

**Table 2:** The standardized mean differences between the TARE and Combo groups before and after the inverse propensity weighting. Note: MELD, Model for End-Stage Liver Disease; BCLC, Barcelona Clinic Liver Cancer classification; ECOG, Eastern Cooperative Oncology Group; AFP, serum alpha-fetoprotein levels; PVTT, portal vein tumor thrombus; TACE, transarterial chemoembolization.

Variable		p-value	Standardized Mean Difference (before adjustment)	Standardized Mean Difference (after adjustment)	
					Sex		0.5051	0.0629	-0.0611	
Age		0.7461	0.0738	0.0338	
Etiology		0.242			
	HCV		-0.1284	0.0387	
	Alcohol		-0.0477	0.0357	
	Cryptogenic		-0.0355	-0.0501	
	NASH		0.0056	0.0009	
	HBV		0.1208	0.0252	
	Other		0.0852	-0.0505	
Cirrhosis		0.1067	-0.1263	0.02	
Child-Pugh		0.4425	-0.0888	-0.0166	
MELD		0.3455	-0.1243	-0.0283	
BCLC		0.01509	-0.2719	0.0523	
ECOG		0.04347	-0.3923	0.0624	
Tumor Type		0.005215			
	Solitary		0.2648	0.0145	
	Multifocal		-0.1857	-0.0241	
	Infiltrative		-0.0791	0.0095	
Tumor Distribution		0.3496	0.1157	-0.0892	
AFP		0.2355	-0.1339	0.0526	
PVTT		0.4786	-0.0832	0.0182	
Extrahepatic Metastasis		>0.99	-0.0041	-0.015	
Prior Treatment		0.04997	-0.2217	-0.2192	
	Prior Surgery	0.7532	-0.0396	-0.0729	
	Prior Ablation	0.03598	-0.1821	-0.1458	
	Prior Chemotherapy	>0.99	-0.0218	-0.0425	
Additional Treatment		0.003984	0.3323	-0.0013	
	Additional Ablation	>0.99	-0.0178	-0.0247	
	Additional Surgery	>0.99	-0.0274	-0.0203	
	Additional Chemotherapy	0.08077	0.14	0.0068	
	Additional TACE	0.0008	0.3115	0.0604	
Sorafenib Use		0.359	0.0852	0.0917	

Thirty-one patients had received prior curative or palliative intent treatment (27 patients in the TARE group vs. four patients in the Combo group). Five patients received microwave ablation as an additional treatment (four in the TARE group vs. one in the Combo group). At the time of data analysis, 72 patients had died, 6 were lost to follow-up, and 22 were still alive.

Treatment outcomes and patient survival

**Figure 1:**
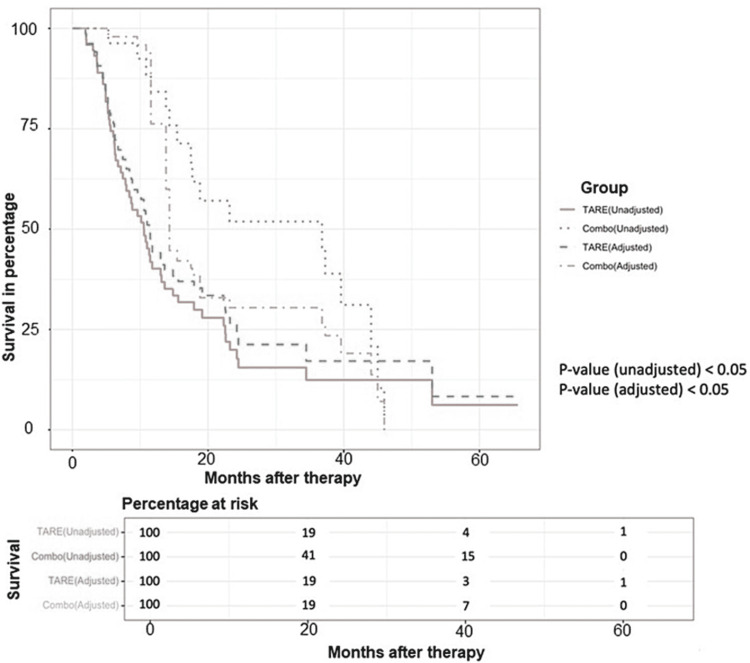
Kaplan-Meier curve illustrates the difference in overall survival (OS) between the Combo and TARE groups before and after the inverse propensity weighting adjustment.

**Figure 2:**
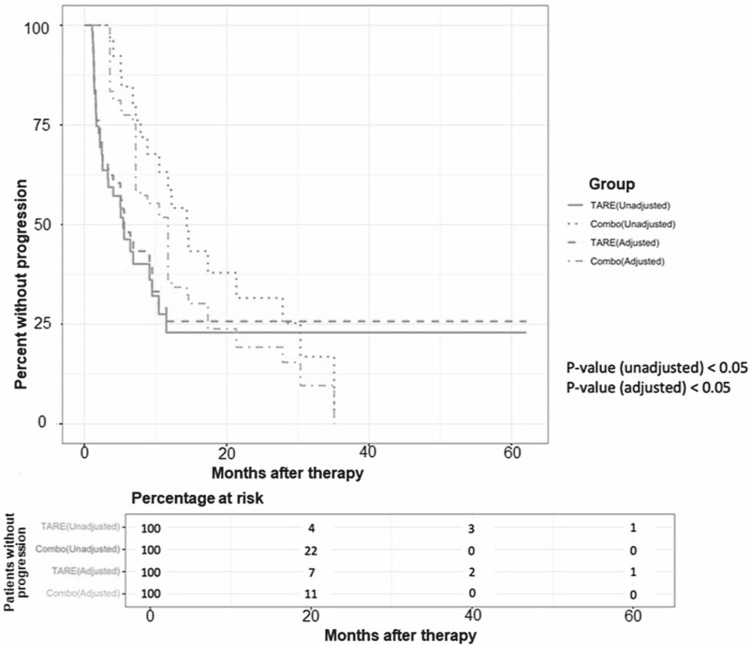
Kaplan-Meier curve illustrates the difference in time to progression (TTP) between the Combo and TARE groups before and after the inverse propensity weighting adjustment.

The median OS of all patients was 11.04 months. Patients in the Combo group had a significantly longer median OS of 36.8 months (vs. 10.6 months, p=0.003) and a median TTP of 14.4 months (vs. 5.5 months, p=0.018) compared to the TARE group. The common confounders (demographic variables, prior and additional treatment information) were incorporated through a multivariate logistic model with inverse propensity weighting, and the selection bias between the Combo group and TARE group was adjusted. The standardized mean differences before and after the adjustment can be found in Table 2. The estimated coefficients with 95% confidence intervals in propensity score estimation are demonstrated in Table 3. Before the adjustment, the hazard ratios of the Combo group and TARE group were 0.441 (p<0.05) for OS and 0.497 (p<0.05) for TTP. After the adjustment, the weighted Cox proportional hazard model was applied to analyze OS and TTP for the treatment groups, which showed the following hazard ratios for the Combo group/TARE group: OS, 0.651 (p<0.05) and TTP, 0.63 (p<0.05). Figures 1, 2 demonstrate OS and TTP comparing the two groups before and after the inverse propensity weighting adjustment.

**Table 3:** The estimated coefficients with 95% confidence intervals in propensity score estimation. Note: MELD, Model for End-Stage Liver Disease; BCLC, Barcelona Clinic Liver Cancer classification; ECOG, Eastern Cooperative Oncology Group; AFP, serum alpha-fetoprotein levels; PVTT, portal vein tumor thrombus; TACE, transarterial chemoembolization.

Variable		Coefficient Estimate (95%- Confidence Interval)	
			Sex		0.2893 [-1.883,2.870]	
Etiology			
	HCV	0.4177 [-1.342,2.254]	
	Cryptogenic	-0.2741 [-2.962,2.013]	
	NASH	0.02855 [-2.564,2.407]	
	HBV	0.7777 [-2.964,5.551]	
Child-Pugh		0.6349 [-1.071,2.399]	
MELD		-0.1114 [-1.683,1.405]	
BCLC		-0.9806 [-3.344,1.215]	
ECOG		-0.2164 [-2.060,1.698]	
Tumor Type			
	Solitary	2.749 [0.2867,5.483]	
	Multifocal	0.7409 [-1.358,2.939]	
AFP		-0.3330 [-2.348, 1.543]	
PVTT		0.9614 [-1.165,3.158]	
Extrahepatic Metastasis		2.554 [-2.514,7.367]	
Prior Treatment			
	Prior Surgery	2.329 [-1.389,7.268]	
	Prior Chemotherapy	0.9032 [-2.787, 5.219]	
Additional Treatment			
	Additional Ablation	-1.817 [-6.684,1.733]	
	Additional Chemotherapy	1.032 [-2.935,6.150]	
	Additional TACE	0.6040 [-3.722, 4.990]	
Sorafenib Use		0.06735 [-3.507,3.065]	

In the Combo group, the time interval between TARE and TACE was less than three months in fourteen (52%) patients who received TACE for incomplete response and more than three months in 13 (48%) patients who underwent TACE for disease progression. TACE procedures were also assigned as early versus late according to the standard follow-up imaging interval after TARE, which is three months. There was no significant difference in OS (p = 0.27) or TTP (p = 0.85) when early (three months) versus late (3+ months) TACE addition was compared.

An assessment of baseline characteristics as prognostic factors was carried out on the whole patient cohort. Patients with a MELD score of ≤9 had a significantly better OS than patients with a MELD score of >9 (p=0.038). Also, higher baseline AFP values (>400 ng/ml) were significantly correlated with a worse OS (p=0.0076).

**Figure 3:**
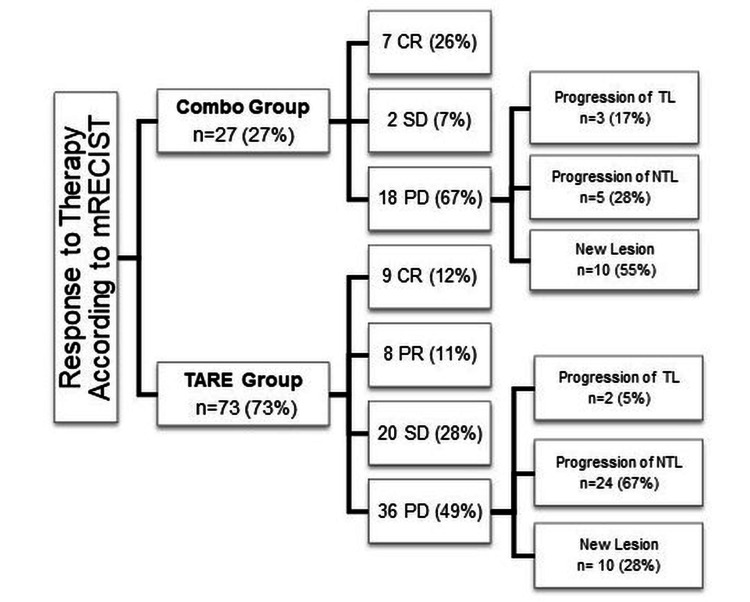
The tumor response and progression reasons based on Modified Response Evaluation Criteria in Solid Tumors (mRECIST) for the whole patient cohort. CR: Complete response, PR: Partial response, SD: Stable disease, PD: Progressive disease, TL: Target lesion, NTL: Non-target lesion.

The tumor response to treatment was evaluated with mRECIST criteria until the last follow-up imaging, and the response of tumors with the most common patterns of disease progression was demonstrated in Figure 3. The disease progression reasons were different in the two treatment groups (p=0.013).

Clinical and laboratory toxicity

Based on the change in bilirubin levels after treatment, more patients in the TARE group had worsening liver function than in the Combo group (40% vs. 19%; p=0.029). Worsening liver function based on the two or more points increase in CP score was observed in 32 (51%) patients in the TARE group and seven (28%) patients in the Combo group (p=0.056). Worsening liver function was observed most commonly (59%) in the first three months.

Major adverse events and complications after radioembolization in both groups are shown in Table 4.

**Table 4:** Complications after radioembolization procedures in TARE and Combo groups according to Society of Interventional Radiology (SIR) Classification System for complications by outcome. Grade A complication requires no therapy with no consequence. Grade C complication requires therapy with minor hospitalization (<48 hours). Grade D complication requires major therapy, unplanned increase in level of care, and prolonged hospitalization (>48 hours).

SIR Grading for Procedural Complications	TARE group n=73 (%)	Combo group n=27 (%)
Grade A	69 (95%)	27 (100%)
Grade C	3 (4%)	-
One large transudative pleural effusion		
One femoral artery pseudoaneurysm		
One puncture site (groin) hematoma		
Grade D	1 (1%)	-
Acute liver failure		

As for the complications of TACE procedures in the Combo group, four patients were hospitalized for 48 hours due to extended post-embolization syndrome, while the remaining 23 were discharged either on the same day or stayed overnight for observation. Most patients experienced fever, abdominal pain, nausea, and vomiting in the first week after TACE as self-limited symptoms.

## Discussion

Our results demonstrate that patients with unresectable HCC who underwent radioembolization followed by TACE had a longer OS and TTP when compared to patients who underwent radioembolization alone. The reason for the longer OS and TTP of the patients in the Combo group may be a possible synergistic or additive effect from the different mechanisms of TACE and TARE. The time interval between TARE and TACE did not seem to impact prognosis. In other words, it did not matter whether subsequent TACE was done early to treat incomplete responses or after the patients progressed.

A selection bias between the two groups was observed in favor of the Combo group which included a higher rate of patients with BCLC stage B, ECOG score 0, and solitary tumors. This variation between two different patient populations can be due to the bias of clinicians when deciding on the appropriate therapy. However, after taking into account this selection bias with a weighted Cox proportional hazards model, the addition of TACE was still found to be associated with longer OS and TTP. In addition, the Combo group had a higher percentage of patients with complete tumor response, although the follow-up period in the Combo group was longer than in the TARE group.

In our study, the addition of TACE was not associated with a higher rate of liver toxicity in the Combo group. A selection bias for earlier stage patients with a good liver function who can tolerate a combination treatment could be why the Combo group had less liver toxicity. In fact, regarding the toxicity assessment based on the bilirubin levels after treatment, a higher number of patients in the TARE group were observed to have increased bilirubin levels. This might be because TARE was given as salvage therapy in some patients in the TARE group, resulting in higher rates of liver failure. Overall, though, the addition of TACE to TARE was not associated with increased liver toxicity.

Considering that tumor progression is a complex process that involves genetic divergence and evolutionary adaptation, and HCC can display intratumoral heterogeneity, disease control can be difficult with one treatment modality targeting only certain aspects of the tumor environment [18,19]. Therefore, consideration can be given to combining certain treatments targeting multiple aspects of the tumor environment and replication mechanisms in patients who can tolerate them. 

Radioembolization and TACE have different mechanisms of action for tumoricidal and hepatotoxic effects. While conventional TACE involves an injection of an ordinary chemotherapeutic drug such as doxorubicin or cisplatin and subsequent embolization of the hepatic artery, which aims to obstruct the vascular lumen, resulting in ischemia and necrosis of the tumor, TARE is a minimally embolic treatment option, and its effects are predominantly due to the radiation carried by microspheres rather than ischemia [6,20]. Therefore, TARE does not preclude any additional transarterial therapies.

There are large meta-analyses that report superior survival outcomes with a combination of TACE and external radiotherapy over TACE alone [9,21,22]. However, external radiation treatment may not be feasible because of relative intolerance to radiation of the liver parenchyma, patient discomfort, and multiple treatment sessions [23]. On the other hand, TARE with Yttrium-90 is considered preferable because radiation can be delivered to hypervascular hepatic tumors in a single treatment session via feeding hepatic arteries while avoiding most of the harmful effects of radiation on the liver parenchyma [23]. TARE is also reported to have reduced toxicity compared to TACE, making TARE more suitable for combination and salvage therapies [24-27]. Here, we investigated the possible additive/synergistic effects of combining radioembolization with TACE since radioembolization does not preclude any additional transarterial therapy due to its minimally embolic nature [6]. This study has further demonstrated that better outcomes were obtained from combining TACE with radiation, in this case, with radioembolization as a more feasible treatment alternative.

As to the limitations, this study was conducted retrospectively with a modest-sized patient population. Also, a selection bias was detected between the two treatment groups, which might have influenced the observed difference in TTP and OS. However, further statistical analysis was performed using a weighted Cox proportional hazards model with propensity score weighting to account for this imbalance between the two groups. When the differences in TTP and OS were assessed while accounting for the selection bias, the addition of TACE to TARE was still found to have a positive effect on TTP and OS.

## Conclusions

Patients with unresectable HCC who underwent radioembolization followed by TACE had a longer overall survival and TTP when compared to patients who underwent radioembolization alone. The addition of TACE was not associated with a higher rate of liver toxicity. Therefore, in select patients, consideration should be given to adding TACE to the treatment protocol for patients undergoing TARE for HCC. Further prospective studies or randomized controlled trials are warranted to confirm better outcomes with TARE and TACE combination therapy compared to TARE alone.
